# Robustness of individual differences in temporal interference effects

**DOI:** 10.1371/journal.pone.0202345

**Published:** 2018-08-14

**Authors:** Nadine Schlichting, Ritske de Jong, Hedderik van Rijn

**Affiliations:** 1 Department of Experimental Psychology, University of Groningen, Groningen, The Netherlands; 2 Research School of Behavioural and Cognitive Neurosciences, University of Groningen, Groningen, The Netherlands; Tokai University, JAPAN

## Abstract

Magnitudes or quantities of the different dimensions that define a stimulus (e.g., space, speed or numerosity) influence the perceived duration of that stimulus, a phenomenon known as (temporal) interference effects. This complicates studying the neurobiological foundation of the perception of time, as any signatures of temporal processing are tainted by interfering dimensions. In earlier work, in which judgements on either time or numerosity were made while EEG was recorded, we used Maximum Likelihood Estimation (MLE) to estimate, for each participant separately, the influence of temporal and numerical information on making duration or numerosity judgements. We found large individual differences in the estimated magnitudes, but ML-estimates allowed us to partial out interference effects. However, for such analyses, it is essential that estimates are meaningful and stable. Therefore, in the current study, we examined the reliability of the MLE procedure by comparing the interference magnitudes estimated in two sessions, spread a week apart. In addition to the standard paradigm, we also presented task variants in which the interfering dimension was manipulated, to assess which aspects of the numerosity dimension exert the largest influence on temporal processing. The results indicate that individual interference magnitudes are stable, both between sessions and over tasks. Further, the ML-estimates of the time-numerosity judgement tasks were predictive of performance in a standard temporal judgement task. Thus, how much temporal information participants use in time estimations tasks seems to be a stable trait that can be captured with the MLE procedure. ML-estimates are, however, not predictive of performance in other interference-tasks, here operationalized by a numerical Stroop task. Taken together, the MLE procedure is a reliable tool to quantify individual differences in magnitude interference effects and can therefore reliably inform the analysis of neuroimaging data when contrasts are needed between the accumulation of a temporal and an interfering dimension.

## Introduction

Our subjective experience of the duration of an event is influenced by concurrent magnitude information of the very same event or stimulus. Examples of these temporal magnitude interference effects on time are the effect of space [1–4], numerosity [3,5–7], or numerical magnitudes [3,8–10]. Usually ‘more’ in the non-time dimension leads to an increased likelihood of ‘longer’ judgements in the time dimension. While such interference effects on duration judgements may be instrumental to understanding the links between temporal cognition and other psychological processes [11], they represent a significant problem or nuisance in research that seeks to elucidate the neural underpinnings of duration estimation. That is, in these tasks participants are often asked to either estimate the duration of a stimulus and ignore the other magnitude, or, vice versa, to estimate the other magnitude and ignore the duration. By means of comparing the differences in neural signatures of both estimations, researchers aim to identify which neural signals are specific to timing. Thus, these interference effects work against the primary goal and rationale of the methods employed in such research: to isolate the neural systems and mechanisms involved in duration estimation by using suitable tasks and appropriate control conditions.

Several considerations or desiderata pertain to a proper design of a neuroimaging study of duration estimation. First, the process at issue can be conceptualized as one in which information about a quantity (here time) accumulates from stimulus onset to offset, with the accumulated total corresponding to the duration judgment. While accumulation of information over time is inherent to temporal judgements, it is not for other stimulus dimensions. To establish specificity with respect to temporal magnitude, a matching task is required in which participants estimate and report the accumulated total with regard to another stimulus dimension, such as dominant color (e.g., [12,13]) or the total distance a dot travelled (e.g., [14]). To ensure similarity to the timing task and sustained attention to the stimulus, the non-temporal dimension should also be presented dynamically over time, so that information has to be accumulated from stimulus onset to offset. Second, to equate the two tasks in terms of sensory input, stimuli in both tasks should contain both the temporal and the non-temporal dimension, thus conveying both relevant and irrelevant magnitude information in both tasks or conditions. A clear contrast between the two tasks would be obtained only if participants were able to selectively attend and process only the relevant stimulus dimension. However, as the behaviorally well-established magnitude interference effects demonstrate, participants tend to process both relevant and irrelevant magnitude information in both conditions, rendering a comparison between the two tasks inferentially problematic and less informative.

In this paper, we describe and test the reliability of a Maximum Likelihood Estimation (MLE) procedure that can potentially disentangle the processing of temporal and other magnitudes at the level of individual participants. Its results suggest that there are strong and seemingly stable individual differences in the size of temporal magnitude interference effects, with a sizable subset of participants apparently being quite capable of selectively attending to relevant magnitude information only. We will discuss how such results can significantly inform and strengthen analyses in neuroimaging studies of duration estimation.

**Fig 1:**
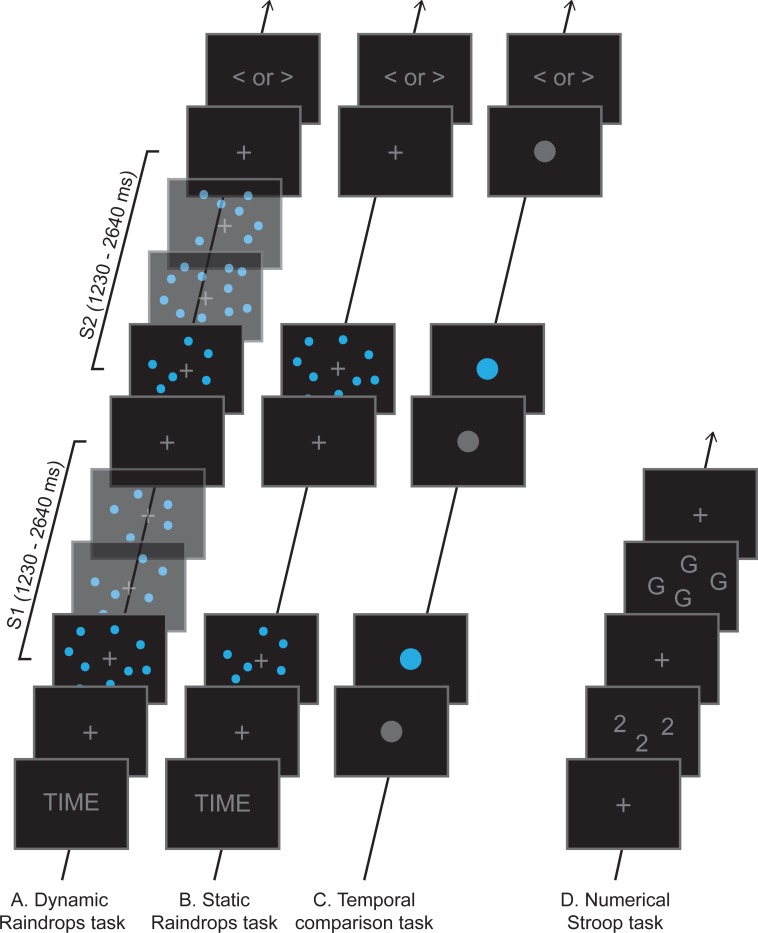
Schematic depiction of task designs. **(A, B)** In a comparison task participants had to judge whether the second stimulus was longer or shorter (*time* dimension) or consisted of fewer or more dots (*number* dimension) than the first stimulus. Participants were cued before blocks of eight trials which dimension would be the target dimension for the next trials. Stimuli consisted of clouds of small blue dots which appeared and disappeared dynamically on the screen (Dynamic Raindrops, panel A) or stayed on screen for the whole interval (Static Raindrops, panel B). Either the first or second stimulus was always the standard stimulus, lasting for 1800 ms and consisting of 30 dots in total, while the other stimulus could take on one of six comparison magnitudes in both dimensions. **(C)** Here, participants only had to make a judgement based on time. Intervals were marked by a grey circle changing color to blue and back to grey. The same durations as in the Raindrops tasks were used in the temporal comparison task. **(D)** In the numerical Stroop task participants had to report how many items were on the screen. Three conditions were employed: 1) congruent (digit magnitude corresponded to number of items), 2) incongruent (digit magnitude did not correspond to number of items), and 3) control (letters). All tasks were self-paced, that is, the next trial only started after a response was given.

In a recent EEG, study we investigated neural signatures of duration estimation compared to numerosity estimation [15]. On each trial, participants saw two consecutive stimuli consisting of a series of blue dots dynamically appearing and disappearing on a black screen, together forming a cloud of dots. Each stimulus was characterized by its duration and the total number of dots it contained (see Fig 1A for a schematic depiction). Because of its visual appearance, we refer to this task as the Dynamic Raindrops task. Participants were asked to judge whether the second stimulus appeared for a shorter or longer duration (time condition) or consisted of fewer or more dots (numerosity condition). Replicating previous findings (see [11] for a review), the behavioral data indicated that judgements on time were significantly affected by numerosity, whereas numerosity judgements were relatively resilient to interference effects. Upon closer inspection, we found large individual differences in the extent to which participants showed interference effects, that is, how strongly irrelevant magnitude information affected their decisions. In order to quantify individual strength of interference effects we used a MLE procedure to estimate, per participant and condition (i.e., time and numerosity), how much each dimension was taken into account when making a decision. The output of the procedure are two parameters or weights, *ω*_time_ and *ω*_number_, which represent the weights by which time and number information contribute to the overall evidence in favor of one or the other response alternative. For example, a good ‘timing’ participant (i.e., a participant who shows little or no interfering effect of numerosity on time) would have a high *ω*_time_ and a low *ω*_number_ in the time condition. Based on the estimated weights, we categorized participants into a group showing only little or no interference effects and a group showing stronger interference effects to guide analysis of the EEG data, with the idea that it is in particular the data of the first group, whose members were apparently quite capable of following instructions to selective attend only the relevant magnitude dimension, that would be more likely to reveal dimension-specific neural differences. In fact, inclusion of the second group, as in more traditional analyses across all participants, might dilute and potentially even obscure such differences. Note that the weights are estimates of the true underlying weights, that is, they are subject to noise because of, for example, limitations in experimental design (e.g., number of trials).

Thus, for a performance-based procedure as described above to yield meaningful and reliable results, the ML-estimated weights should provide reasonably accurate and reliable estimates of the true underlying weights for each individual participant. This can, for example, be attained by including a sufficient number of trials [16]. More interestingly, the procedure would be most generally useful and powerful if true underlying weights, and their empirical estimates, are stable over time. From a conceptual perspective, individual differences in the size of temporal magnitude interference effects would seem most interesting if these represented a relatively stable trait. From a practical perspective, such stability is necessary when aggregating data over multiple sessions or when selecting participants for subsequent neuroimaging studies based on the results of behavioral screening sessions. Such stability of test results over time is typically referred to as test-retest reliability. Recently, several authors have drawn attention to the fact that test-retest reliabilities of many popular and well-established cognitive tasks in psychology and neuroscience are surprisingly low, given their common use. Hedge, Powell, and Sumner [17] describe the statistical issues associated with well-established tasks often having poor reliabilities as the reliability paradox: The very reason that such tasks produce robust and easily replicable effects–low between-individual variability–also tends to cause low reliabilities of these effects, making their use as correlational tools problematic. These authors also suggested that these statistical issues, while having a long history in psychology, tend to be widely overlooked in cognitive psychology and neuroscience today (for related concerns, results, and possible remedies, see [16,18,19]). Thus, the first aim of the present study was therefore to empirically establish the test-retest reliability of the ML-estimated weights for time and number information in the Dynamic Raindrop task, using two sessions separated by six to eight days.

The second aim of the present study was to shed more light on the nature of the use of temporal and numerical information when making comparative temporal judgments in the Dynamic Raindrops task. As explained earlier, to allow for a properly matched task, evidence for the non-temporal magnitude should also accumulate over time. However, this introduces an emergent and highly salient visual feature, the rate of appearance/disappearance of raindrops. Even though rate is not necessarily constant over the presentation of one stimulus due to the raindrops’ random onsets, here we will focus on the average rate during stimulus duration. Given the short life time of individual drops and their randomized onsets, this average rate is closely associated with the average number of raindrops visible during stimulus duration. Note that the average rate for a stimulus also corresponds to the total number of raindrops divided by stimulus duration. This means that interference effects on temporal judgments might be based on numerosity, on average rate, or on a combination of these two potential factors. In the experiments reported here, stimulus numerosity and duration were quasi-randomly combined so as to keep the negative and positive correlation of average rate with duration and numerosity close to -.5 and .5 respectively (but at a cost of thereby introducing a .5 correlation between duration and numerosity; see [15] and Methods for details). Because of the simple mathematical relationship between duration, numerosity and average rate the MLE procedure is not able to differentiate between these three factors and models exhibit mimicry behavior. However, strong reliance on rate information in the Dynamic task version will have marked effects on the estimated weights for temporal and numerical information: For instance, suppose that in the Dynamic Raindrops task numerosity has an interfering effect on temporal judgments (i.e., ‘more’ leads to an increased likelihood of ‘longer’ judgments); and suppose further that also average rate has an effect on temporal judgments (as average rate will be strongly negatively correlated with duration across trials and positively correlated with numerosity, higher average rate can be expected to lead to a decreased likelihood of ‘longer’ judgments). As rate is positively correlated with numerosity, but these factors have opposite interfering effects on temporal judgment, their combined effects tend to cancel out and could mask interference effects.

In the present study, we used two different versions of a Static version of the Raindrops task (Fig 1B) to assess the possible usage of rate information in temporal judgments in the Dynamic Raindrops version, based on the following rationale: When rate information is taken away, as in the Static Raindrops task, this should give rise to marked changes in these estimated weights, resulting in relatively low correlations between Dynamic and Static tasks. Therefore, in the first session, we included a Static version of the Raindrops task with the .5 correlation between numerosity and duration left intact. In the second session, we included a Static version but now with the correlation between numerosity and duration removed–to the extent that this correlation affected performance in the Dynamic Raindrops task, its removal should be expected to yield even lower correlations. The comparison of the two versions of the Static Raindrops task will shed additional light on whether the artificially introduced correlations between time and numerosity affected participants markedly in the way they incorporate task-relevant and task-irrelevant information.

Additionally, we were interested in the generalizability to other, non-magnitude tasks. For this purpose, we tested the relation between *ω*_time_ estimates in the Raindrops tasks and level of performance in a timing task without any interfering dimension (Fig 1C). Further, we tested the relation between temporal magnitude interference effects to other well-established interference effects, here Stroop interference in a numerical Stroop task (Fig 1D). We chose the numerical Stroop task because it produces relatively stable Stroop effects compared to the colour-word Stroop task [20–22].

All results reported and discussed here are based on data from the time condition, which is the condition of main interest in the literature discussed earlier, and the focus of our earlier work. Moreover, we observe more pronounced interference effects in this condition. However, all scripts to run the experiments and analyses, all data, as well as analyses and results based on the number condition can be found online at osf.io/b73u2.

## Materials and methods

### Ethics statement

The research was conducted in accordance with the Declaration of Helsinki; the Ethical Committee Psychology of the University of Groningen approved the experiments and procedures (identification number 16218-S-NE). Participants gave written informed consent prior to testing.

### Participants

Fifty-six participants enrolled in the Bachelor program Psychology at the University of Groningen participated in the experiment in exchange for course credits. Due to technical problems, data of seven participants was not saved correctly, and thus discarded. A further eight participants were excluded from the analysis because of too varying or suboptimal performance suggesting non-compliance with instructions. Specifically, exclusion was based on four performance measures, derived from a logistic function fitted to each participants data using the Psignifit toolbox version 3.0 for Matlab [23]: participants were excluded 1) if their Weber Ratio (computed as half the distance between values that support 25 and 75% of "longer" ("more") responses normalized by the Point of Subjective Equality), averaged over all tasks, was larger than 0.5, 2) if the standard deviation of the Weber Ratios over all tasks was larger than 0.4 (hinting at a large variability in performance), 3) if the difference between the highest and lowest Weber Ratio was larger than 1, and 4) if the proportion of correct responses in relatively easy trials was lower than in relatively difficult trials in more than one task. The final sample comprised data of 41 participants (25 female) aged between 18 and 26 years (*M* = 20.27 years).

### Stimuli and tasks

All stimuli and tasks were created using Matlab 7.13 (The MathWorks) and the Psychophysics toolbox version 3.0.12 [24] running under Windows 7 (version 6.1) and displayed on a 1280 × 1024 CRT-monitor screen with a refresh rate of 100 Hz.

### Dynamic Raindrops task

In a comparison task participants had to judge whether the second stimulus (S2) presented in a trial was shorter or longer (*time* dimension) or consisted of fewer or more dots (*number* dimension) than the first stimulus (S1), whereby either S1 or S2 was always the standard stimulus (Fig 1). Participants were cued in advance whether they had to make a judgement on *time* or on *number*.

Clouds of blue dots served as stimuli (RGB: 0, 0, 255). Each cloud consisted of single dots that appeared and disappeared dynamically on a black screen. The duration of each stimulus was marked by the appearance of the first dot (onset) and disappearance of the last dot (offset). The number of dots was determined by the total number of dots presented. Each stimulus could vary simultaneously and independently in *time* and *number*.

The lifetime of each dot (i.e., the interval between appearance and disappearance of the dot) was sampled from a uniform distribution between 400 and 800 ms. Multiple dots could be visible at the same time, and it was ensured that at least one dot would be on screen during the interval. Dots had a radius of 2.5 px and appeared within a virtual ring with an outer radius of 150 px and an inner radius of 50 px around the fixation cross. Positions of single dots within one trial were chosen randomly, with the constraint that dots could not overlap in space (i.e., they were separated by at least 10 px). The standard stimulus was set to a duration of 1800 ms and to consist of 30 dots in both *time* and *number* trials. The probe stimuli in both dimensions took six possible magnitude values defined as 1.1^−4^, 1.1^−2^, 1.1^−1^, 1.1^1^, 1.1^2^ and 1.1^4^ times the standard magnitude (to ensure precise presentations timing, durations were rounded to the second and number of dots was rounded to the nearest integer), resulting in durations of 1230, 1490, 1630, 1980, 2180 and 2640 ms, and 20, 25, 27, 33, 36, and 44 dots. Probe stimuli can be further categorized as congruent (i.e., both dimensions vary in the same direction, e.g., shorter and fewer dots) and incongruent (i.e., dimensions vary in different directions, e.g., shorter and more dots).

A consequence of the dynamic nature of this experimental design is that both the task-irrelevant dimension (i.e., *number* in *time*-trials and *time* in *number*-trials) as well as the rate of drop appearance (i.e., how quickly drops appear and disappear) can be predictive of the task-relevant dimension. For example, during the presentation of T_1_N_6_ the maximum number of dots would appear during the shortest duration so that the rate of dots appearing would be very fast. To limit predictiveness of the task-irrelevant dimension and the rate of dot appearance on the dimension to be judged, the task-irrelevant magnitude was chosen randomly from a weighted uniform distribution. Weights were 0.8 for the same magnitude as the task-relevant magnitude, and 0.75, 0.55, 0.25, 0.05 and 0 for magnitudes with increasing distance from the task-relevant magnitude (hence, the shortest duration will not be paired with the largest number of dots). Using these weights, we simulated 10,000 stimuli and found a correlation between time and numerosity of r = .51 (i.e., how well does one magnitude predict the other magnitude), and a correlation of r = .50 between time/numerosity (r = -.47 for time) and rate of drop appearance (i.e., how well does drop appearance rate predict the other magnitudes). These correlations show that with the selected weights we can ensure that the task-irrelevant dimension and rate of drop appearance are equally predictive of the task-relevant dimension. A major problem in trying to eliminate one of the correlations (e.g., between time and numerosity) is that the other factor (following the example, rate) becomes highly predictive of the attended dimension. For example, randomly sampling the task-irrelevant magnitude without any weights results in a very low correlation of r < .01 between time and numerosity, but rate becomes predictive of both time (r = -.66) and number (r = .70). Thus, as described above, we determined the task-irrelevant magnitude using weighted random sampling to make both the task-irrelevant and rate of drop appearance equally predictive of the task-relevant magnitude. The script running this simulation and additional ones using different ways to combine task-relevant and task-irrelevant magnitudes can be found online at osf.io/b73u2. The task design is identical to the task previously used in an EEG experiment (see [15]).

The experiment was divided into two blocks, each block consisting of 80 trials. Within each block, *time* and *number* trials were alternating in sub-blocks of eight trials each. The order of these sub-blocks was counterbalanced between participants. Before each sub-block, participants were cued whether they had to make a judgement on *time* or on *number*. In each block, in half of the *time-*trials the standard stimulus was presented as S1, in the other half it was presented as S2. The probe stimulus in each of the two conditions (standard stimulus as S1 or S2) was longer than the standard duration in half of the trials, and shorter in the other half. Out of the 40 *time* trials in each block, the two most extreme probe durations (1230 and 2640 ms) were presented four times each, while all other probe durations were presented eight times each. The same logic was true for *number*-trials.

Each trial started with the presentation of a grey fixation cross for a duration sampled from a uniform distribution between 800 and 1200 ms. Then, S1 and S2 were presented consecutively with an inter-stimulus-interval sampled from a uniform distribution between 1200 and 1600 ms. The fixation cross remained on screen for another 800–1200 ms before the response screen appeared and stayed until a response was given. Participants were instructed to press ‘S’ on a conventional US-Qwerty keyboard if they perceived S2 as shorter or consisting of fewer dots than S1, and ‘L’ if they perceived S2 as longer or consisting of more dots than S1. A blank screen appeared for 800–1200 ms before the next trial started (see Fig 1 for a visual depiction of an experimental trial). After half of the trials, participants could take a self-timed break. Participants received feedback on their performance (percentage correct trials) during the break and after completion.

### Static Raindrops tasks

The static versions of the Raindrops task are essentially like the Dynamic Raindrops task. The only and crucial difference was that the drops did not appear dynamically on the screen, but all dots appeared at stimulus onset and disappeared at stimulus offset (see Fig 1). We designed two versions of the Static Raindrops task, differing in how the task-irrelevant dimension was sampled with respect to the task-relevant dimension. We used a correlated version, which used the same constraints as the Dynamic Raindrops task, and an uncorrelated version, in which the task-irrelevant dimension was sampled randomly without any constraints. The latter resulted in a correlation between time and number magnitudes of r < .01, meaning that the task-irrelevant dimension is not at all predictive of the task-relevant dimension.

### Temporal comparison task

In the temporal comparison task participants only had to make a judgement based on time. Again, the same durations and response formats as in the Raindrops tasks were used. Instead of showing multiple small dots, participants saw one bigger dot with a fixed size (radius = 25 px). The first interval (S1) was marked by the dot changing its color from grey to blue (onset) and back to grey (offset), the second interval (S2) was presented in the same way (see Fig 1). After half of the trials, participants could take a self-timed break. As for the Raindrops tasks, participants received feedback on their performance (percentage correct trials) during the break and after completion.

### Numerical Stroop task

Three to six white colored digits (i.e., the same digit, ranging from 3 to 6) or letters (A, F, K or P) appeared within a circle (radius = 120 px) around the center of the black screen. Characters had a size of 15 × 24 px and had a minimum distance of 50 px to other characters. Participant’s task was to report how many items appeared on the screen by pressing the appropriate digit-key. Participants were instructed to place their left middle and index finger on the keys ‘3’ and ‘4’, and their right index and middle finger on keys ‘5’ and ‘6’ at all times. The numerical Stroop experiment had three conditions: 1) congruent (i.e., the digit magnitude corresponded to the number of items), 2) incongruent (i.e., the digit magnitude did not correspond to the number of items), and 3) control condition (letters instead of digits). In total, participants completed 304 trials: 108 incongruent trials (each digit appeared nine times in each number of items condition), 100 congruent trials (each digit appeared 25 times in its number of items condition), and 96 control trials (each letter appeared 6 times in each number of items condition). Trials of all conditions were presented in randomized order. The next trial started after a response was given. The inter-trial-interval was sampled from a uniform distribution ranging from 600 to 1000 ms. After half of the trials, participants could take a break for as long as they liked. No feedback on performance was given to the participants.

### Procedure

Participants were tested in two sessions separated by six to eight days. In session one, participants completed the Dynamical Raindrops task (Dynamic I), followed by the numerical Stroop task, and, at the end of session one, the correlated version of the Static Raindrops task (Static I). During session two, participants again started with the Dynamic Raindrops task (Dynamic II), followed by the temporal comparison task, and ended with the uncorrelated version of the Static Raindrops task (Static II). Each session took approximately 75 minutes.

### Data analysis

#### MLE procedure

To quantify how strongly participants took numerical and temporal evidence into account when making a judgement on either dimension in the Raindrops tasks, we estimated these two parameters using a Maximum-Likelihood Estimation (MLE) procedure (for another example of the application of this MLE procedure, see [15]). The underlying model used the weighted sum of temporal and numerical evidence for each trial (*evidence*_*total*_, see Eq 1), that is, parameter estimation was stimulus driven. Temporal and numerical evidence (*evidence*_*time*_ and *evidence*_*number*_, respectively) was determined by subtracting the magnitudes of the standard stimulus from the magnitudes of the non-standard stimulus and subsequently dividing by the maximal evidence possible, so that the estimate was scaled from -1 to 1 (i.e., the more different the non-standard stimulus magnitudes were from the standard stimulus magnitudes, the more *evidence* was available in a given trial, see Eqs 2 and 3 for an example of the time condition).

$${evidence}_{total}=\omega_{time}\times {evidence}_{time}+\omega_{number}\times {evidence}_{number}$$ (1)

$${evidence}_{time}=\frac{T_{comparison}-T_{s}}{maximum\;evidence\;possible}$$ (2)

$${maximum\;evidence\;possible}_{time}=\left\{ \begin{array}{c} T_{S}-T_{1}=0.57,if\;T_{comparison}<T_{s}\\ T_{6}-T_{S}=0.84,if\;T_{comparison}>T_{s} \end{array}\right.$$ (3)

**Fig 2:**
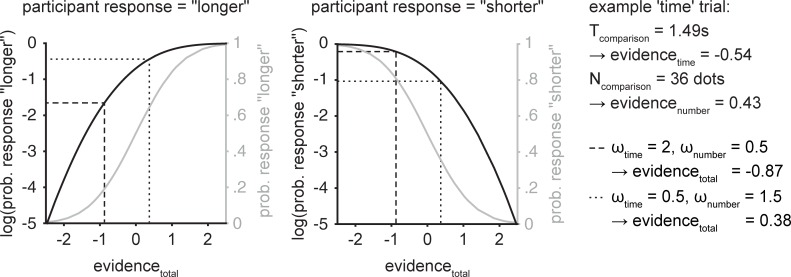
Graphical illustration and numerical example of the MLE procedure. For each of the 80 trials evidence_total_ was calculated based on the stimulus parameters and the weights selected during each iteration of the MLE procedure (Formula 1). Evidence_total_ was then used to compute the cumulative probability of a response (“longer”, grey curve left panel; “shorter”, grey curve right panel). In each iteration of the MLE procedure and for each trial the logarithm of the probability of the participant’s actual response (response “longer”, black curve left panel; response “shorter”, black curve right panel) given was computed with the current weights. The final weights were those for which the sum of the log-values over all trials was maximal. Dashed and dotted lines show two examples of different sets of weights and their effects on the weight-selection process on one specific incongruent trial. In the dotted line, more numerosity information was used, thus this set of weights would be superior if participant’s response was “longer” (i.e., influenced by the incongruent numerosity information and reflected in a higher log-value). On the contrary, the dashed line is an example of a set of weights in which temporal information is taken into account more than numerosity information. Here, if the participant correctly responds “shorter”, this set of weights will be favoured (higher log-value). Note however, that the weights selection was based on all trials.

*Evidence*_*number*_ and *maximum evidence possible* based on numerosity were calculated according to the same principles. The weights *ω*_time_ and *ω*_number_ were estimated in the MLE procedure. *Evidence*_*total*_ was then used to compute the probability of a response (shorter/fewer or longer/more) based on a standard normal cumulative distribution (see Fig 2, grey curves). The final weights were those for which the sum of the logarithms of the probability of a specific response were maximal (i.e., the weights that best predicted behavioral data on a trial by trial basis). For a visual depiction and a numerical example see Fig 2. Using this procedure, we obtained a weight for time and a weight for number for each Raindrops task, condition and participant. The reported model, including both time and number information, outperformed models including only time or only number information (comparisons can be found online at osf.io/b73u2).

To obtain a comparable estimate of how much temporal evidence was used in the non-magnitude temporal comparison task, we estimated *ω*_time_ for the temporal comparison task in a model using only temporal evidence.

#### Vector correlations

In order to investigate the robustness of individual differences in the usage of time versus numerosity information in timing performance, as represented by *ω*_time_ and *ω*_number_ respectively, we combined the two estimates into a vector (i.e., *ω*_time_ is treated as the x-component and *ω*_number_ as the y-component) and calculated vector correlations between all different versions and sessions of the Raindrops task. Vector correlations convey information about the relatedness of two vector fields [25–27]. The output of this vector correlation comprises the correlation coefficient *p* ranging from 0 (no correlation) to 1 (perfect correlation), a rotation angle θ and a scaling factor β, describing the amount of rotation and scaling needed to best align the two vector fields (for more detailed information and formulas, see [25]). Hanson et al. [25] distinguish between rotational and reflectional correlations, however, because we do not expect to find a reflectional relationship between vector fields, we decided a-priori to only calculate vector correlations based on rotational dependencies. Notably, if the variance of the two vector fields is very similar, scale factor β is very similar to *p*. In the current data set, we found similar variance in all tasks (i.e., β values provide no additional information). Further, we found little evidence for a systematic rotation of vector fields between tasks (i.e., rotation angle θ of around zero). Thus, we will only report the correlation coefficients *p*. Values of rotation angle θ and scale factor β can be found online at osf.io/b73u2. For all empirical correlations 95% confidence intervals were calculated using nonparametric bootstrapping [28].

#### Relation to temporal comparison and Stroop task

In order to test whether being a ‘timer’ in the Raindrops task (i.e., having a comparably high *ω*_time_) is related to performance in the non-magnitude temporal comparison task, *ω*_time_ parameters obtained from the different Raindrops tasks were correlated with *ω*_time_ parameters obtained from the temporal comparison task.

The Stroop effect is calculated for each participant and is defined as the median reaction time in the incongruent condition subtracted by the median reaction time in the congruent condition. This score was correlated with *ω*_number_ obtained in each version of the Raindrops task, because *ω*_number_ reflects the amount of interference in the time condition of the Raindrops tasks.

For all empirical correlations 95% confidence intervals were calculated using nonparametric bootstrapping.

## Results

### Stability of magnitude interference effects over time

**Fig 3:**
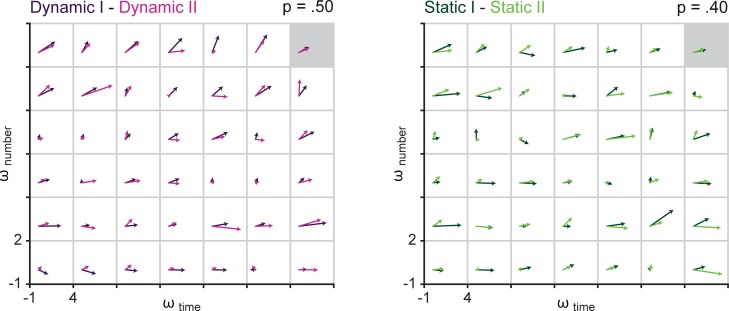
Vector correlations assessing stability of interference effects over time. Vector fields show the composed *ω*-vectors (*ω*_time_ as x-component and *ω*_number_ as y-component) for each participant (i.e., each square, distribution is constant over all panels) and the grand average (grey highlighted square). Correlations were computed between *ω*-vectors of session I and session II in the Dynamic Raindrops task (left hand side) and in the Static Raindrops tasks (right hand side).

**Fig 4:**
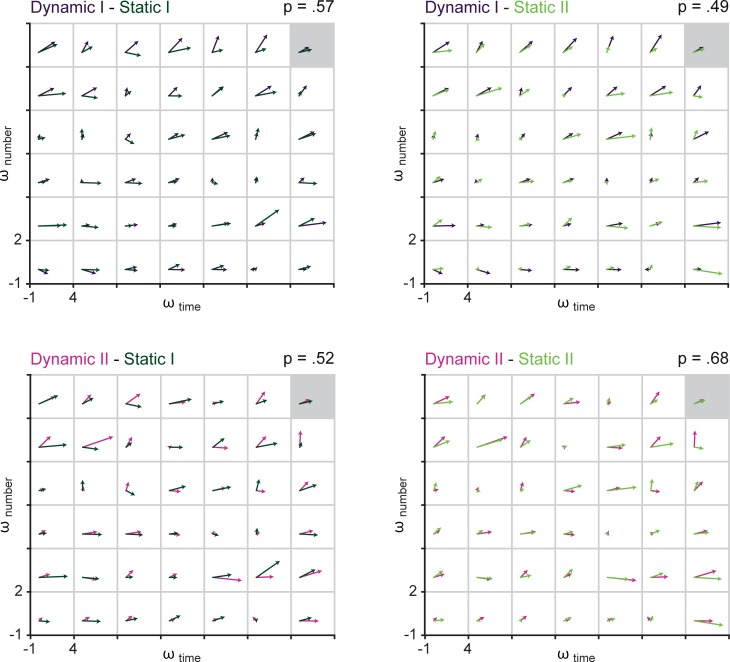
Vector correlations assessing stability of interference effects over tasks (and time). Vector fields show the composed *ω*-vectors (*ω*_time_ as x-component and *ω*_number_ as y-component) for each participant (i.e., each square, distribution is constant over all panels) and the grand average (grey highlighted square). Correlations were computed between *ω*-vectors of different versions of the Raindrops task.

For each condition in each of the Raindrops tasks we obtained *ω*_time_ and *ω*_number_ as output of the MLE procedure. These weights are estimates of how much temporal and numerical evidence participants took into account when making a judgement on time (as reported here). The estimated weights of each task can be regarded as vectors, with *ω*_time_ treated as the x-component and *ω*_number_ as the y-component. This way, a unique field of vectors is obtained for each task (i.e., one vector for each participant in each task, see also Figs 3 and 4). We calculated vector correlations to assess the relatedness of vector fields between Raindrops tasks performed in session one and in session two (i.e., Dynamic I versus Dynamic II and Static I/correlated versus Static II/uncorrelated). We employed a vector correlation method advanced by Hanson, Klink, Matsuura, Robeson, and Willmott [25], which was initially developed for the analysis of geographic data, but has been applied to neuroscientific data, too (e.g., [26]). The output of this vector correlation method comprises, among other parameters, the correlation coefficient *p*, ranging from 0 (no correlation) to 1 (perfect correlation). Nonparametric bootstrapping [28] was used to calculate 95% confidence intervals.

The empirical correlations between the Dynamic Raindrops task performed in session one and two (*p* = .50, 95% CI [.29 .66]) and between the Static Raindrops task performed in session one and two (*p* = .40, 95% CI [.22 .57]) are shown in Fig 3. In both tasks, a positive correlation is found, suggesting relative stability of the *ω*-estimates over sessions.

### Stability of magnitude interference effects over task

To test the stability of interference effects (quantified by the *ω*-estimates) over different tasks vector correlations as described above were calculated. Here, we correlated *ω*-estimates obtained from the Dynamic Raindrops tasks with those obtained from Static Raindrops tasks. Notably, two of the correlations are based on tasks performed in the same session (i.e., Dynamic I versus Static I and Dynamic II versus Static II), while the other two are based on tasks performed in different sessions (i.e., Dynamic I versus Static II and Dynamic II versus Static I). Results of empirical correlations are summarized in Fig 4. Tasks that were run in the same session are correlated highly (Dynamic I—Static I: *p* = .57, 95% CI [.35 .75]; Dynamic II—Static II: *p* = .68, 95% CI [.52 .80]). For tasks that were performed in different sessions, empirical correlations are numerically lower, however, 95% confidence intervals are still far removed from zero (Dynamic I—Static II: *p* = .49, 95% CI [.27 .68]; Dynamic II—Static I: *p* = .52, 95% CI [.36 .67]).

### Comparison to performance in temporal comparison task

**Fig 5:**
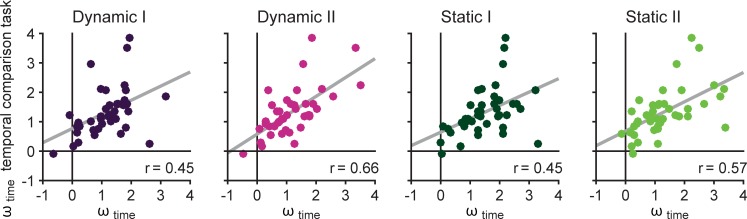
Correlations between timing performance in temporal comparison task and magnitude comparison tasks. Testing whether being a ‘timing’ participant in a magnitude comparison task (quantified by *ω*_time_) is correlated to performance in a comparison task which only has the time dimension and no other interfering information of a different dimension. Each dot represents one participant; grey line shows the regression line.

In order to have a similar performance measure in the temporal comparison task compared to the Raindrops tasks, we calculated *ω*_time_ also for the temporal comparison task. For this purpose, the model underlying the MLE procedure incorporated only *ω*_time_. The *ω*_time_ parameters obtained from the different Raindrops tasks were then correlated with *ω*_time_ parameters obtained from the temporal comparison task. 95% confidence intervals were calculated for all correlation coefficients by using nonparametric bootstrapping [28]. Fig 5 shows that all tested pairs yield a high correlation (Dynamic I: *r* = .45, 95% CI [.13 .70]; Dynamic II: *r* = .66, 95% CI [.45 .81]; Static I: *r* = .45, 95% CI [.15 .69]; Static II: *r* = .57, 95% CI [.36 .74]). Further, as all 95% confidence intervals do not contain zero, being a ‘timer’ in a magnitude interference tasks (i.e., having a high *ω*_time_), likely means being a ‘timer’ in other timing tasks without interfering influences.

### Comparison to performance in numerical Stroop task

The Stroop effect was calculated for each participant as the median reaction time in the incongruent condition subtracted by the median reaction time in the congruent condition (*M* = 68.96 ms, 95% CI [54.17 83.76] ms). This score was then correlated with *ω*_number_ as an index of interference in the time condition of the Raindrops tasks. 95% confidence intervals were calculated for all correlation coefficients by using nonparametric bootstrapping [28].

**Fig 6:**
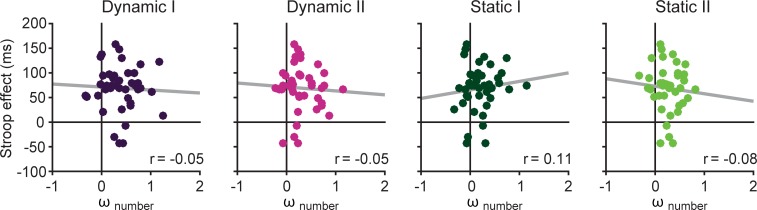
Correlations between Stroop effect and magnitude interference effects. To test whether participants showing larger magnitude interference effects (quantified by *ω*_number_) also show larger Stroop interference effects, these two scores were correlated for each task. Each dot represents one participant; grey line shows the regression line.

Results, visually summarized in Fig 6, show that correlations are very low and all 95% confidence intervals contain zero (Dynamic I: *r* = -.05, 95% CI [-.30 .21]; Dynamic II: *r* = -.05, 95% CI [-.30 .17]; Static I: *r* = .11, 95% CI [-.16 .38]; Static II: *r* = -.08, 95% CI [-.29 .14]). Thus, we failed to find any evidence for, on the one hand, a relation between the degree to which interfering information is incorporated in the Raindrops magnitude tasks, and the magnitude of the numerical Stroop effect on the other.

## Discussion

In the current study, we tested the reliability and stability of a MLE procedure that can potentially serve as a tool to quantify the magnitude of interference effects between time and numerosity at the level of individual participants. Our results replicated earlier work in which we found large individual differences in the magnitude of interference effects. That is, when asked to make a judgement on the time dimension, some participants are influenced by task-irrelevant numerosity information more than others. Extending previous findings, we showed that these individual differences are stable and robust over time and over similar, yet different task versions. This suggests that the ability to ignore or inhibit task-irrelevant information in magnitude comparison tasks could be seen as a ‘stable trait’ or ‘psychological bias’ [29] within participants.

To facilitate interpretation of our findings, we first want to explain in more detail how vector correlations can be interpreted and which possible advantages this procedure brings to the field of cognitive neuroscience, before we discuss the results in more detail.

### Interpretation of vector correlations

**Fig 7:**
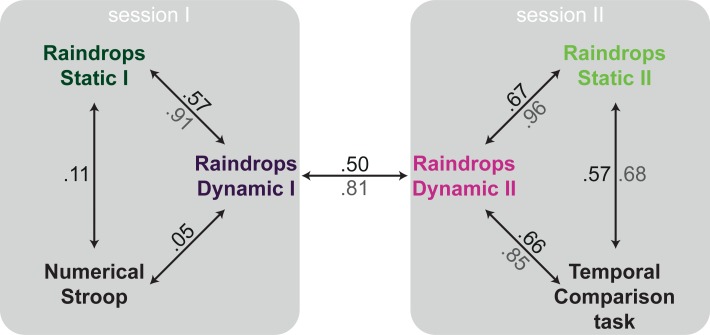
Summary of the main results. Empirical (black) and disattenuated (grey, where relevant) correlations between sessions and versions of the Raindrops task, as well as the Numerical Stroop and Temporal Comparison task. (See main text for additional details).

The calculation of vector correlations enables researchers to assess associations between sets of two-dimensional data (i.e., vector fields). Vector correlations are rarely used in the field of neuroscience or cognitive psychology (but, see [26,27]). Yet, alternative measures of association between multiple two-dimensional data-sets are suboptimal. For example, one could calculate Pearson product-moment correlations or cross-correlations for each dimension separately. However, in direct comparison vector correlations are superior to other correlation analyses because they provide one unified correlation coefficient [27]. Alternatively, one could transform the data to reduce two-dimensional to one-dimensional data. Critically, any such transformation will result in information loss. For example, calculating vector length (or magnitude) does not capture information about vector orientation (i.e., the angle between the vector and the x-axis), and vice versa. Vector correlations, on the other hand, allow for one unified correlation coefficient encapsulating all information available in the data. Fig 7 summarizes the vector correlations (shown in black) observed in the current study.

Importantly, empirically determined correlations are known to be attenuated due to measurement error [17]. Results of Monte Carlo simulations showed that the combination of the number of trials per task (here 80) and the correlation between duration and numerosity resulted in relatively high measurement error, and thus variability in the MLE-determined weights for temporal and numerical information. In other words, the correlations between MLE-determined weights, including test-retest reliabilities, are likely to be markedly weakened by the diluting effects of measurement error. For this reason we have employed standard techniques to correct these empirically estimated correlations for attenuation (e.g., [18,30,31]). In short, we used estimated split-half reliabilities followed by application of the Spearman-Brown prophecy formula to obtain empirical estimated reliabilities of individual parameters. These estimated reliabilities were then used to compute disattenuated estimates of the various correlations, shown in grey in Fig 7. Details of the computational procedure and relevant results are available at osf.io/b73u2. Note that the two correlations with the numerical Stroop task could not be corrected for attenuation because the estimated reliabilities of the two parameters involved–the weight for numerosity information, *ω*_number_, and the size of the Stroop effect–were too small to warrant such a correction. For the remaining correlations, the effect of the correction for attenuation can be seen to be quite substantial. Because these corrected values are more likely to reflect the “true” strength of associations, we will focus on these values in the following discussion of the results.

### Stability of magnitude interference effects over time

When re-tested after seven days in both the Dynamic and the Static version of the Raindrops task (Dynamic I–Dynamic II and Static I–Static II), ML-estimates exhibited considerable test-retest reliability (see Fig 7). It should be noted that the two versions of the Static Raindrops task differed in the way the magnitude of the task-irrelevant dimension was sampled (using restricted random sampling or random sampling): While during the first session the task-irrelevant magnitude was a limited predictor of the task-relevant magnitude (restricted random sampling), it was not predictive during the second session (random sampling). That is, in the Static Raindrops I task participants could have based their temporal judgements on numerosity information and still perform reasonably well in the task. Importantly, irrespective of the nature of the numerosity dimension (i.e., static/dynamic or correlated/uncorrelated), robust test-retest correlations were observed.

### Stability of magnitude interference effects over task versions

ML-estimates were also robust with regard to the different versions of the Raindrops task, irrespective of whether they were performed during the same or different sessions. ML-estimates of tasks that were performed within the same session (Dynamic I–Static I and Dynamic II–Static II) in fact yielded very high correlations, as shown in Fig 7. In light of previous findings, the commonality between Dynamic and Static versions of the Raindrops tasks with regard to the magnitude of interference as captured by ML-estimates is noteworthy for two reasons:

First, an often reported finding when using static numerosity-time comparison tasks is that temporal judgements are influenced by task-irrelevant numerosity information, but not vice versa ([3,7]; but see Javadi & Aichelburg [32], who found bidirectional interference effects). In contrast, Lambrechts, Walsh and van Wassenhove [33] and Martin, Wiener and van Wassenhove [34] found that when time, space and number information are presented *dynamically*, duration judgments are resilient to spatial and numerical interference, while time influences judgments of the other two dimensions. In the current and in a previous study [15], we found that direction and magnitude of interference effects in static and dynamic setups vary greatly between participants, but are stable over task versions within participants. Given that both findings have been replicated in independent studies, it is unlikely that these contrasting results are driven by participant sampling even though the observed variability of ML-estimates suggests that sampling effects might influence the outcomes of interference studies. Even though the paradigms used are similar, drawing decisive conclusions is precluded by small differences in the task setup. To resolve this paradox, future work should present both paradigms in a within-subject design.

Second, it has been argued that experimentally testing interference effects is complicated by the fact that manipulating one dimension will alter other stimulus dimensions, too (e.g., see [35] for an extensive discussion for the case of automatically co-varying space, density, and/or surface when manipulating numerosity). In the case of time and numerosity, presenting numerosity information dynamically over time introduces the additional dimension rate of change or rate of sensory evidence accumulation [33,34], which can, if not controlled for, be highly predictive of the task-relevant dimension. In the current study, we controlled the predictiveness of rate information by restricting which values or magnitude levels the task-relevant and -irrelevant dimension could take on. However, theoretically, stimuli in the dynamic version of the Raindrops task contain more predictive information than stimuli in the static versions: both the task-irrelevant dimension as well as rate information is, to a certain degree, predictive of the dimension to be judged. The current results show that ML-estimates capturing interference effects are stable over dynamic and static versions of the Raindrops task, again suggesting that mechanisms underlying interference effects may be considered as a stable trait, and suggesting that rate did not influence participants’ judgements strongly.

In a way, the temporal comparison task can be regarded as a control task for *time* trials in the Raindrops tasks, because stimuli contain no interfering numerosity information. The way participants utilized temporal information in the most straight forward task (temporal comparison task) is predictive of how temporal information are utilized in the presence of interfering information (Raindrops tasks). It may seem striking that for some participants the ML-estimate was close to zero, however, *timing* is a very noisy process in comparison to other magnitude tasks (e.g., compared to number and length judgements [36]).

### Generalizability to Stroop interference task

The ability to ignore or inhibit task-irrelevant information in any version of the Raindrops task was not predictive of performance in a numerical Stroop task. An interpretation of these “null” results is that magnitude interference effects adhere to different inhibitory control mechanisms than Stroop-like interference effects: Interference effects in magnitude comparison tasks could be governed by bottom-up or stimulus-driven processes, while interference in Stroop task could be driven more by top-down processes, given that the interfering information first needs to be semantically parsed (i.e., the meaning of a digit or a color-word, see also van Maanen, van Rijn and Borst [37], who argue that semantic interference effects are caused by the same interference mechanism). Furthermore, Stroop-type interference has been found to yield only moderate test-retest correlations and very limited parallel-test stability [17,21,22], meaning that scores in Stroop task are relatively noisy and unstable, again indicating that one should be careful drawing firm conclusions.

### Conclusion

Especially for neuroimaging studies relying on task-setups that include a well matched comparison task (similar to the Raindrops tasks tested here), it is crucial to take into account that 1) participants exhibit interference effects and apparently do not only process task-relevant information, and that 2) the magnitude of how much of the task-irrelevant information is used in making a judgement differs between participants. Also for theoretical and computational models explaining the processing of magnitudes (e.g., A Theory Of Magnitudes [38,39]; or Bayesian approaches to quantify magnitude interference effects [34,40]) it is important to know whether the magnitude of interference is a stable trait within participants, or whether it can change over time or due to modified task designs. The here described MLE procedure allows to account for these individual differences in behavior. We showed that the MLE procedure is a reliable tool to quantify individual differences in temporal interference effects in terms of test-retest and parallel-test reliability. How well participants can estimate time and how much task-irrelevant information they take into account seems to be a stable characteristic within individuals. As patterns of individual differences in temporal magnitude interference effects, here captured by vector correlations which take into account all available information, are stable over time and over task versions, the methods presented here provide a valuable addition to the toolkit of cognitive neuroscientists interested in studying the processing of different stimulus dimensions.

While the here proposed MLE procedure is designed for quite specific experiments in which stimuli vary in magnitude in two or more dimensions, we argue that, more generally, our results emphasize the potential information gain from quantifying inter-individual differences [29,41] and highlight the importance of establishing reliability of parameters derived from individual behavior or behavioral performance before their subsequent use in neuroimaging analyses.
